# Perception and Knowledge of Hospital Workers Toward Using Artificial Intelligence: A Descriptive Study

**DOI:** 10.1002/hsr2.70623

**Published:** 2025-04-30

**Authors:** Ahmad R. Al‐Qudimat, Mohammad R. Alqudimat, Kalpana Singh, Zainab E. Fares, Mohammed Ismail, Hesham E. Yasin, Raed M. Al‐Zoubi, Omar M. Aboumarzouk

**Affiliations:** ^1^ Surgical Research Section, Department of Surgery Hamad Medical Corporation Doha Qatar; ^2^ Department of Public Health, QU‐Health, College of Health Sciences Qatar University Doha Qatar; ^3^ American University of the Middle East Kuwait City Kuwait; ^4^ Department of Nursing Hamad Medical Corporation Doha Qatar; ^5^ Department of Biomedical Sciences, College of Health Sciences QU‐Health, Qatar University Doha Qatar; ^6^ Department of Biomedical Sciences, College of Health Sciences Qatar University Doha Qatar; ^7^ Department of Chemistry Jordan University of Science and Technology Irbid Jordan; ^8^ School of Medicine, Dentistry, and Nursing The University of Glasgow Glasgow UK

**Keywords:** healthcare workers, knowledge, perception, using artificial intelligence

## Abstract

**Objective:**

This study aims to assess the perception and knowledge of hospital workers toward using artificial intelligence (AI) in healthcare in Qatar, exploring the potential implications of AI integration in clinical settings.

**Methods:**

A descriptive cross‐sectional study was conducted from January 1, 2023, to January 31, 2024, involving healthcare workers (HCWs) from various departments across four leading hospitals affiliated with Hamad Medical Corporation (HMC) in Qatar. A convenience sampling method was used to recruit participants, and data were collected via an anonymous online questionnaire distributed through email and WhatsApp, with hard copies provided as needed. The questionnaire covered demographic information, perception, and knowledge of AI. The data were analyzed using STATA 17.0, with significance set at *p* < 0.05.

**Results:**

The study included 902 HCWs, with a gender distribution of 47.6% female and 52.4% male. Most participants were from Asia (50.8%), with 42.7% aged between 35 and 44 years. Most participants were nurses (54.9%), followed by allied health professionals (20.7%), pharmacists (12.2%), and physicians (11.0%). A significant portion of participants (59.8%) reported no prior experience with AI. However, 56.1% felt somewhat comfortable using AI in medical practice, and 72% believed that AI would improve the future of healthcare. Despite this optimism, 81.7% had not yet utilized AI in their practice, though 85.4% expressed a desire to learn more about AI models.

**Conclusion:**

The study reveals a positive perception and moderate knowledge of AI among HCWs in Qatar. However, the actual use of AI in clinical practice remains limited. There is a clear need for targeted educational programs to enhance AI proficiency and confidence among healthcare professionals, potentially improving patient outcomes and clinical efficiency. Further research should explore the barriers to AI adoption and develop strategies to facilitate its integration into healthcare systems.

## 1 Introduction

The first use of artificial intelligence (AI) can be traced back to the early 1970s. Edward Shortliffe at Stanford University developed one of the first AI systems in clinical settings to help identify blood infections and treatments [1]. Since then, AI has emerged as a game‐changing technology with the potential to revolutionize industries, including healthcare. The use of AI in healthcare has gathered attention in recent years because of its ability to enhance the delivery of healthcare services [2]. AI‐based tools are anticipated to become part of healthcare services soon, uncovering new applications in radiology, pathology, ophthalmology, oncology, and clinical decision support [3, 4, 5]. One of the advantages of incorporating AI into healthcare is the potential to improve patient outcomes. AI‐powered diagnostic tools can be accurate and efficient, making timely treatment decisions [2, 3, 6]. For example, AI algorithms can effectively analyze images with remarkable precision and identify abnormalities, which can assist radiologists in making accurate diagnoses [3]. In addition to this capability, AI is promising for risk prediction and aiding robotic surgery procedures and automated imaging diagnosis. Additionally, it can contribute significantly to advancements in research [3, 4].

Using AI in healthcare introduces ethical challenges, encompassing privacy protection, addressing biases and discrimination, ensuring transparency and accountability, defining responsibilities and liabilities, obtaining consent, maintaining human interaction, and preserving empathy within care delivery [1]. These ethical considerations highlight the complexity of integrating AI technologies into healthcare systems and the need for comprehensive strategies to mitigate potential risks. For example, it is crucial to establish guidelines to ensure responsible and ethical use of AI while promoting transparency [1, 7]. Healthcare leaders should develop frameworks and policies for utilizing AI in healthcare. Regulatory agencies should collaborate with healthcare institutions to define standards and evaluation protocols that guarantee AI's safe and effective implementation in healthcare. Regular audits, monitoring systems, and reporting mechanisms can be employed to assess the safety, quality assurance aspects, and ethical considerations related to AI‐based services [2].

Educating healthcare workers (HCWs) and the public is crucial to enhancing AI proficiency. Researchers, HCWs, and the public should grasp AI's mechanics and potential strengths and weaknesses [8]. Programs focused on education and training can help bridge the knowledge gap and ensure AI is used effectively in healthcare. Research has shown that HCWs believe that AI has the potential to enhance efficiency, accuracy, and overall outcomes in healthcare practices [9]. A recent cross‐sectional online survey done in Germany found that just 25.2% of nurses can be classified as AI experts, revealing a large gap in AI literacy (Sommer et al., 2024). This study emphasizes the need for expanded AI education and training among nursing workers to bridge the knowledge gap [10]. Also, HCWs view AI as a tool that supplements their skills and improves patient care [11], and they can use AI to rapidly analyze data and identify patterns that might not be easily discernible by humans alone [12]. HCWs also acknowledge the potential of AI in aiding decision‐making processes like drug development [12].

However, there are concerns among HCWs regarding the application of AI in healthcare. Additionally, concerns persist regarding potential errors or biases within AI algorithms that could impact patient safety [11]. They also raise concerns about errors or biases within AI algorithms that could significantly affect patient care [13]. Skepticism regarding AI's reliability, particularly among HCWs who have encountered technology‐related errors, further complicates adoption [9]. Privacy and data security are also key concerns [14].

While various studies have examined AI attitudes among HCWs worldwide, there has been little research into how HCWs and non‐HCWs in Qatar perceive AI integration in healthcare. The existing literature focuses mostly on AI's technological capabilities and ethical considerations, but research on healthcare personnel's attitudes, worries, and preparedness to use AI is limited, particularly in the Middle Eastern environment. This study seeks to fill this vacuum by looking into the viewpoints of hospital workers in Qatar—both HCWs and non‐HCWs—on AI utilization. This study gives useful insights into key issues, perceived benefits, and hurdles to AI adoption, which may be used to influence legislation and training programs to encourage AI integration in the region. Therefore, the research question guiding this study is: What are the viewpoints of hospital workers (HCWs and non‐HCWs) in Qatar regarding the utilization of AI, and what are the perceived benefits, challenges, and key issues influencing AI adoption in the healthcare sector?

## 2 Methodology

This descriptive, cross‐sectional study was conducted from January 1, 2023, to January 31, 2024. The study involved HCWs from various departments, including doctors, nurses, pharmacists, laboratory technicians, and others, working across different departments, such as surgery, ICU, and informatics, at four leading hospitals (Hamad General Hospital [HGH], Women's Wellness and Research Center [WWRC], Al Wakra Hospital, and Hazm Mebaireek General Hospital [HMGH]) affiliated with Hamad Medical Corporation (HMC) in Qatar.

### 2.1 Sample Size

We utilized a convenience sampling method to determine the sample size using the formula:

$$n=\frac{Z^{2}P(1-p)}{d^{2}}$$

In this formula, *Z* represents the *z*‐score corresponding to the desired confidence level (for instance, 1.96 for a 95% confidence level), and *p* denotes the estimated proportion of the population possessing the attribute of interest. When this proportion is unknown, 0.5 is often used, as it provides the maximum sample size. The complement of *p*, or 1 − *p*, reflects the proportion of the population that does not have the attribute of interest. Lastly, *d* indicates the estimate's desired margin of error (precision), typically expressed as a decimal (e.g., 0.05 for ±5%). Accordingly, the minimum recommended sample size was calculated to be 500 participants.

We used a convenience sample strategy since it is practicable and feasible for reaching a varied population of healthcare professionals across several departments and hospitals. This strategy enabled us to efficiently recruit volunteers within the study period. However, we agree that convenience sampling may induce biases, such as selection bias and restricted generalizability. Because participants were chosen based on their availability and willingness to participate, the sample may not reflect the entire population of HCWs in Qatar. To address this constraint, we sought to involve individuals from a variety of hospitals and professional backgrounds to broaden the range of opinions [15]. Furthermore, while our findings give useful insights, they should be evaluated in light of the sample limits, and future studies should use random sampling techniques to improve generalizability.

### 2.2 Eligible Criteria

The inclusion criteria encompassed hospital staff, including healthcare professionals (such as physicians, nurses, and pharmacists) and non‐HCWs (such as those in IT, health informatics, and engineering) working in designated hospitals. Eligible participants were required to have at least a bachelor's degree, possess a minimum of 1 year of professional experience, and demonstrate willingness to participate. Exclusion criteria were applied to those who declined participation.

### 2.3 Questionnaire

We developed a questionnaire based on the review of the literature [2, 6, 7, 14, 16, 17, 18, 19]. The initial domain delves into demographic characteristics and background information, encompassing aspects such as gender, nationality, age, specialty, and others, employing a series of multiple‐choice questions. The second section centers around perception, while the third section delves into knowledge. The knowledge base section of the survey was structured to focus on participants' experiences with AI, beliefs regarding ChatGPT, AI usage, current benefits of AI, perspectives on AI's future, barriers to AI application, beliefs about AI, and the desire to utilize AI models.

The questionnaire was created specifically for our study, including features from previously validated surveys while customizing items to the unique objectives of our research. To verify content validity, we examined current research on AI perception among HCWs and tailored relevant items.

We conducted a pilot study involving 20 conveniently selected HWCs and non‐HCWs, in which we discussed with them the comprehensiveness, language, and grammar of the questions. To assess the face and content validity of the questionnaire, we distributed it to four reviewers, consisting of two health professionals and two senior researchers. Each reviewer was asked to independently rate each item in the questionnaire and provide feedback on its readability, comprehensiveness, clarity, language, and grammar. Test–retest reliability was assessed utilizing *κ* coefficients with a threshold set at ≥ 0.4. The Cronbach's *α* coefficient for the scale employed in the present study yielded a robust value of 0.87.

### 2.4 Description of Data Collection Tool

Data collection took place utilizing an anonymous online questionnaire. The e‐questionnaire was created using Microsoft Teams forms and Google Docs in English. The questionnaire was then distributed to HCWs via email and WhatsApp. Hard copies were also distributed as a second option in case of accessibility issues for the e‐questionnaires, for example, technical issues. Participation was voluntary and anonymous. Providing consent for using the anonymous data was an integral part of the survey, and participating in the survey constituted consent.

### 2.5 Statistical Analysis

After completing the survey, the data of the participants were transferred to Excel. A rigorous review of all responses was undertaken to validate the accuracy of the data. Absolute frequency and percentage were employed to illustrate categorical variables, while mean and standard deviation were utilized for continuous variables in the depiction. The Fisher's exact test and *χ* ^2^ test were employed to investigate the correlation between professionals and their level of knowledge. The data were analyzed through the STATA 17.0 statistical software, with a significant level of *p* < 0.05 being statistically significant in the context of two‐sided tests.

### 2.6 Ethical Approval

The study followed ethical guidelines outlined by the Declaration of Helsinki, Good Clinical Practice Guidelines, and those established by the Surgical Research Section (SRS) with approval number “SR/RE/2023/68.” Consent was obtained from all participants, who were informed about the study's objectives and assured that all shared information would remain confidential.

## 3 Results

**Figure 1:**
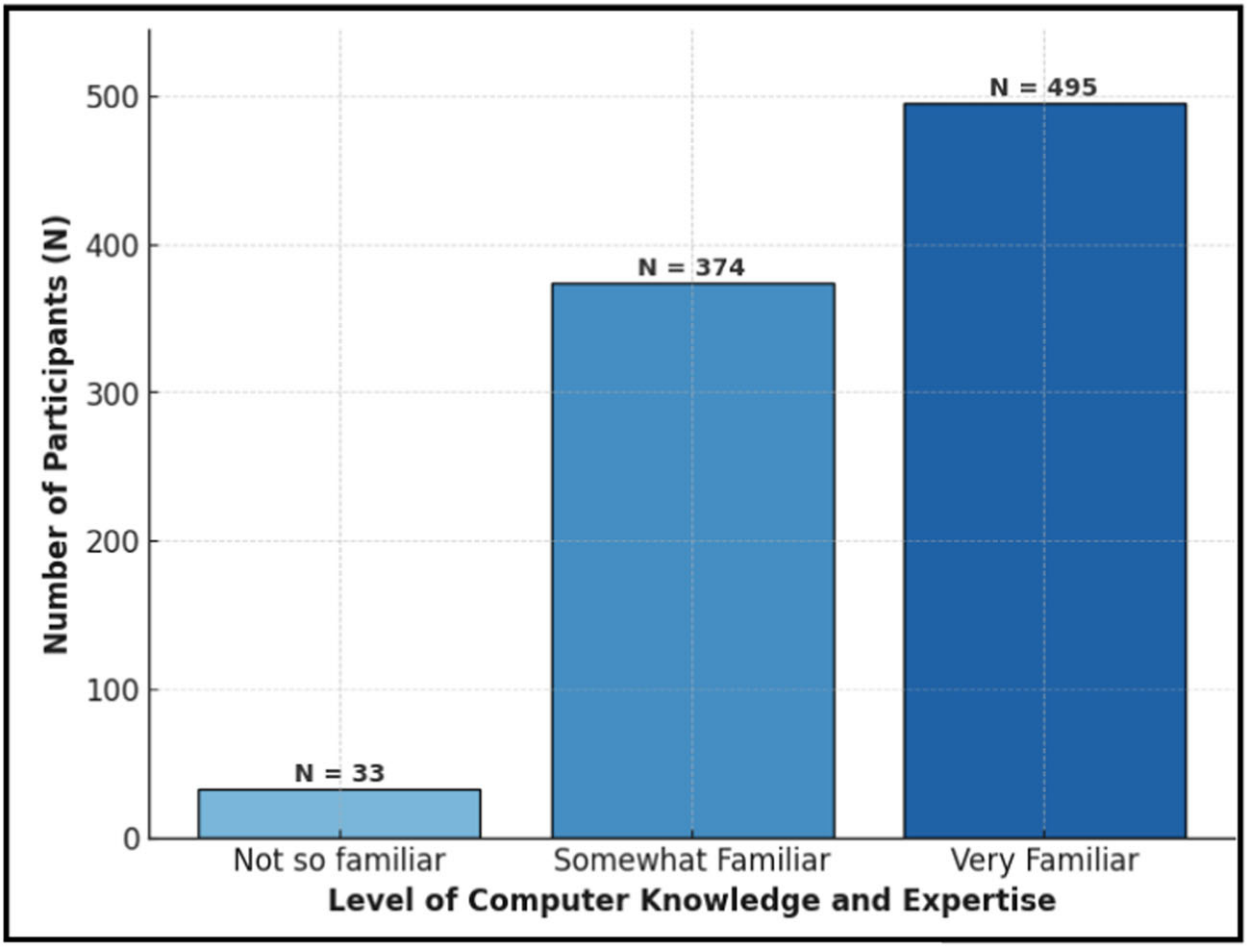
Participants' level of computer knowledge and expertise.

The survey involved 902 HCWs, with 47.6% being female. Among them, 42.7% are aged between 35 and 44 years. The majority, 98.7%, were HCWs, while 1.2% came from healthcare informatics backgrounds. Among them, 54.9% were nurses, which includes clinical nurse specialists, informatics nurses, and general nurses; 20.7% were allied health professionals (dentist, lab technician, public health, and researchers); 11% were physicians, and 12.2% were pharmacists, who used AI in their daily work (see Table 1). The data shows that 28.7% of participants had less than 5 years of experience, 18.8% had worked for 10–15 years, 50% had 10–20 years of experience, and 2.5% had over 20 years of experience in healthcare. Regarding nationality, 2.5% are attributed to North America, 50.8% are from Asia, 4.7% originate from Africa, and 42% did not specify their nationality. Table 1 and Figure 1 show the demographic information of the study sample.

**Table 1:** Demographic characteristics of the participants (*N* = 902).

Variables		*N* (%)
Gender	Male	473 (52.4%)
	Female	429 (47.6%)
Nationality	Asia	453 (50.8%)
	Not specified	374 (42.0%)
	Africa	42 (4.7%)
	North America	22 (2.5%)
Age in years	18–24	99 (11.0%)
	25–34	319 (35.4%)
	35–44	385 (42.7%)
	45–54	77 (8.5%)
	55–64	22 (2.4%)
Specialty	Nurse	495 (54.9%)
	Allied Health Professional	187 (20.7%)
	Pharmacist	110 (12.2%)
	Physician	99 (11.0)
	Engineer	11 (1.2)
Experience in healthcare	10–20 years	440 (50.0%)
	Less than 5 years	253 (28.7%)
	5–10 years	165 (18.8%)
	More than 20 years	22 (2.5%)

### 3.1 Perception of Hospitals Workers About Using AI

**Figure 2:**
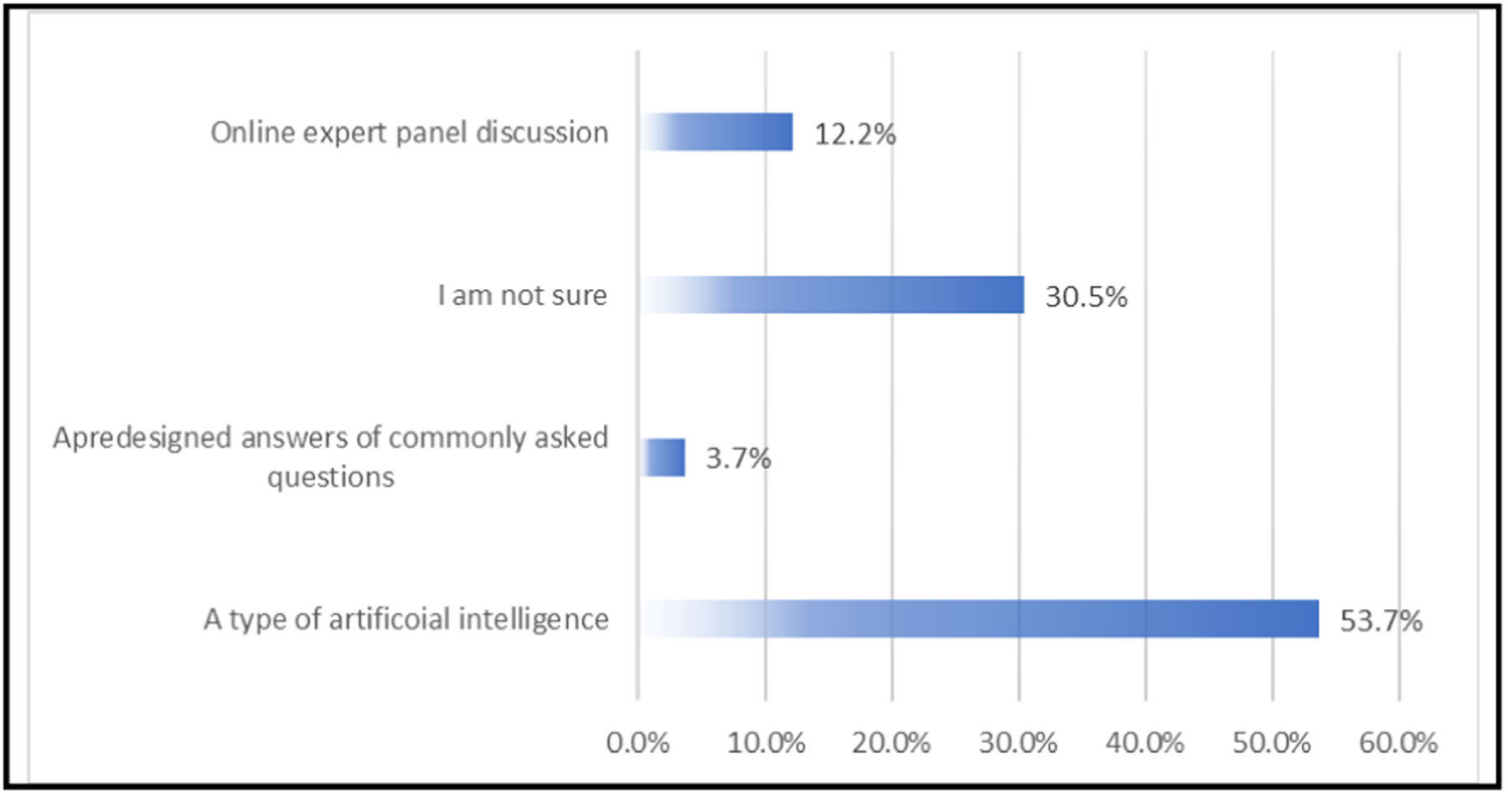
Participants' beliefs about ChatGPT.

Regarding opinions on current AI usage and benefits among participants, the findings indicated that 40.2% currently utilize AI, with 17.1% expressing strong belief in AI and 43.9% holding it as moderate. Regarding belief in ChatGPT, 53.7% recognized it as a form of AI, while 12.2% identified it as an online expert panel discussion, 3.7% perceived it as offering predesigned answers to frequently asked questions, and 30.5% were unsure. In healthcare, participants mainly utilized AI for drafting manuscripts (35.4%) in research, 8.5% utilized it for providing appraisals of literature, 6.1% used it for healthcare decision‐making, and 15.9% used it for providing support to families and patients (Figure 2).

### 3.2 Knowledge Levels Toward Using AI

Concerning the ease of using AI in healthcare practice, 24.4% felt very comfortable, 56.1% reported feeling somewhat comfortable, and 19.5% expressed not feeling comfortable at all. Regarding the potential for AI to improve patient outcomes, 38.3% of participants believed AI would contribute to improving outcomes in the future, 50.6% neither agreed nor disagreed, 9.9% disagreed, and 1.2% were uncertain about its impact. Specifically, regarding AI's future in healthcare, 72% of respondents believed it would enhance healthcare, 6.1% thought it would worsen it, and 22% believed it would have no impact. Furthermore, 81.7% of participants had not utilized AI in their healthcare practice, yet 85.4% expressed an increased desire to learn more about AI models (Table 2).

**Table 2:** Questions related to participants' understanding and experience with AI in healthcare.

Variables	Level	*N* (%)
Any experience with AI	No	539 (59.8%)
	Yes	363 (40.2%)
AI being used in the medical field	Helping in medical research (like drafting manuscripts)	319 (35.4%)
	Providing support to patients and families	143 (15.9%)
	Provide an appraisal of medical literature	77 (8.5%)
	Helping in medical research (like drafting manuscripts); providing appraisal of medical literature	66 (7.3%)
	Providing medical decisions; helping in medical research (like drafting manuscripts); providing appraisal of medical literature	66 (7.3%)
	Providing medical decisions	55 (6.1%)
	Helping in medical research (like drafting manuscripts); providing medical decisions	44 (4.9%)
	Helping in medical research (like drafting manuscripts); providing support to patients and families	33 (3.7%)
	Providing medical decisions; providing support to patients and families	22 (2.4%)
	Providing support to patients and families; helping in medical research (like drafting manuscripts); providing appraisal of the medical literature; providing medical decisions	22 (2.4%)
	Providing support to patients and families; providing an appraisal of the medical literature	22 (2.4%)
	Helping in medical research (like drafting manuscripts); providing support to patients and families; providing appraisal of medical literature	11 (1.2%)
	Provide an appraisal of medical literature, providing support to patients and families	11 (1.2%)
	Providing support to patients and families; providing appraisal of the medical literature; providing medical decisions	11 (1.2%)
Felt ease when using AI in medical practice	Somewhat comfortable	506 (56.1%)
	Very comfortable	220 (24.4%)
	Not comfortable at all	176 (19.5%)
Believe that AI can lead to better patient outcomes	Neither agree nor disagree	451 (50.6%)
	Agree	341 (38.3%)
	Disagree	88 (9.9%)
	I don't know	11 (1.2%)
Potential effects AI have on the future of healthcare	Improve the future of healthcare	649 (72.0%)
	Have no impact	198 (22.0%)
	Worsen the future of healthcare	55 (6.1%)
Not used AI in healthcare practice	Yes	737 (81.7%)
	No	143 (15.9%)
	I don't know	22 (2.4%)
Do you believe that artificial intelligence (AI) should be used to make	A moderate amount	396 (43.9%)
	A little	242 (26.8%)
	A lot	154 (17.1%)
	None at all	110 (12.2%)
Increased your desire to learn more about AI model	Yes	770 (85.4%)
	No	132 (14.6%)

### 3.3 Association of Knowledge Aspect Among Professions

Table 3 presents the correlation between profession‐related knowledge and computer proficiency. Engineers exhibited the highest familiarity with computer knowledge and expertise (100%), surpassing nurses (57.7%), physicians (55.6%), pharmacists (50%), and allied health professionals (47.1%) significantly (*p* < 0.001). Concerning experience with AI, all engineers had exposure to AI compared to physicians (44.4%), nurses (42.2%), pharmacists (20%), and allied health professionals (41.2%) (*p* < 0.001). Notably, nurses (31.1%) expressed more comfort in using AI in practice compared to physicians (22.2%), pharmacists (20%), and allied health professionals (11.8%) (*p* < 0.001). Physicians (77.8%) exhibited greater confidence in the potential positive impact of AI on patient outcomes compared to nurses (40.9%), pharmacists (70%), and allied health professionals (56.3%) (*p* < 0.001). Engineers held stronger beliefs regarding the potential future implications of AI on healthcare compared to nurses (75.6%), physicians (55%), allied health professionals (76.5%), and pharmacists (60%) (*p* < 0.001). The utilization of AI in healthcare practice differed significantly among engineers (100%), nurses (88.4%), physicians (66.7%), pharmacists (80%), and allied health professionals (82.4%) (*p* < 0.001). All professions expressed a heightened interest in learning more about AI models, a statistically significant trend (*p* < 0.001) (Table 3).

**Table 3:** Relationship between various aspects of knowledge and the profession.

	Nurse (*n* = 495)		Pharmacist (*n* = 110)		Allied health professional (*n* = 187)		Physician (*n* = 99)		Engineer (*n* = 11)	
	col %	95% CI	col %	95% CI	col %	95% CI	col %	95% CI	col %	*p* value
Rate your level of computer knowledge and expertise										
Not so familiar (*n* = 33)	4.4	[2.9, 6.7]	0	0	5.9	[3.3, 10.3]	0	0	0	**<** **0.001** *
Somewhat familiar (*n* = 374)	37.8	[33.6, 42.1]	50	[40.7, 59.3]	47.1	[40.0, 54.2]	44.4	[35.0, 54.3]	0	
Very familiar (*n* = 495)	57.8	[53.4, 62.1]	50	[40.7, 59.3]	47.1	[40.0, 54.2]	55.6	[45.7, 65.0]	100%	
Any experience with AI										
No (*n* = 539)	57.8	[53.4, 62.1]	80	[71.5, 86.5]	58.8	[51.6, 65.7]	55.6	[45.7, 65.0]	0	**<** **0.001** *
Yes (*n* = 363)	42.2	[37.9, 46.6]	20	[13.5, 28.5]	41.2	[34.3, 48.4]	44.4	[35.0, 54.3]	100	
At ease would you feel using AI in your medical practice										
Not comfortable at all (*n* = 176)	15.6	[12.6, 19.0]	10	[5.6, 17.2]	23.5	[18.0, 30.2]	44.4	[35.0, 54.3]	0	**<** **0.001** *
Somewhat comfortable (*n* = 506)	53.3	[48.9, 57.7]	70	[60.8, 77.8]	64.7	[57.6, 71.2]	33.3	[24.8, 43.2]	100	
Very comfortable (*n* = 220)	31.1	[27.2, 35.3]	20	[13.5, 28.5]	11.8	[7.9, 17.2]	22.2	[15.1, 31.5]	0	
Believe AI can lead to better patient outcomes										
Agree (*n* = 341)	45.5	[41.1, 49.9]	20	[13.5, 28.5]	37.5	[30.6, 44.9]	22.2	[15.1, 31.5]	100	**<** **0.001** *
Disagree (*n* = 88)	13.6	[10.9, 17.0]	10	[5.6, 17.2]	6.3	[3.5, 10.9]	0	0	0	
Neither agree nor disagree (*n* = 451)	40.9	[36.6, 45.4]	70	[60.8, 77.8]	56.3	[48.8, 63.4]	77.8	[68.5, 84.9]	0	
Potential effects do you see AI to have on the future of healthcare										
Have no impact (*n* = 198)	22.2	[18.8, 26.1]	20	[13.5, 28.5]	11.8	[7.9, 17.2]	44.4	[35.0, 54.3]	0	**<** **0.001** *
Improve the future of healthcare (*n* = 649)	75.6	[71.6, 79.1]	60	[50.6, 68.7]	76.5	[69.8, 82.0]	55.6	[45.7, 65.0]	100	
Worsen the future of healthcare (*n* = 55)	2.2	[1.2, 4.0]	20	[13.5, 28.5]	11.8	[7.9, 17.2]	0		0	
You have not yet used AI in your healthcare practice										
No (*n* = 143)	11.6	[9.0, 14.8]	20	[13.5, 28.5]	17.6	[12.8, 23.8]	33.3	[24.8, 43.2]	0	**<** **0.001** *
Yes (*n* = 737)	88.4	[85.2, 91.0]	80	[71.5, 86.5]	82.4	[76.2, 87.2]	66.7	[56.8, 75.2]	100	
To what extent do you believe artificial intelligence (AI) should be used in making medical decisions for healthcare professionals?										
A little (*n* = 242)	31.1	[27.2, 35.3]	20	[13.5, 28.5]	29.4	[23.3, 36.3]	11.1	[6.3, 19.0]	0	**<** **0.001** *
A lot (*n* = 154)	17.8	[14.6, 21.4]	10	[5.6, 17.2]	23.5	[18.0, 30.2]	11.1	[6.3, 19.0]	0	
A moderate amount (*n* = 396)	44.4	[40.1, 48.9]	50	[40.7, 59.3]	29.4	[23.3, 36.3]	55.6	[45.7, 65.0]	100	
None at all (*n* = 110)	6.7	[4.8, 9.2]	20	[13.5, 28.5]	17.6	[12.8, 23.8]	22.2	[15.1, 31.5]	0	
This survey increased your desire to learn more about AI models										
No (*n* = 132)	13.3	[10.6, 16.6]	10	[5.6, 17.2]	11.8	[7.9, 17.2]	22.2	[15.1, 31.5]	100	**<** **0.001** *
Yes (*n* = 770)	86.7	[83.4, 89.4]	90	[82.8, 94.4]	88.2	[82.8, 92.1]	77.8	[68.5, 84.9]	0	

*Note:* Test statistics: *χ* ^2^ test.

* A *p* value less than 0.05 is considered statistically significant.

Table 4 showed a significantly higher proportion of females (5.1%, 95% CI: [3.4, 7.7]) reported being “Not so familiar” with computers compared to males (2.3%, 95% CI: [1.3, 4.2]), with a *p* value of 0.022. Similarly, AI experience varied significantly by gender, with 71.8% (95% CI: [67.3, 75.9]) of females reporting no AI experience versus 48.8% (95% CI: [44.3, 53.3]) of males (*p* < 0.0001). Comfort with AI in medical practice also differed, as 24.4% (95% CI: [21.7, 27.3]) of participants felt “Very comfortable,” with more males (27.9%, 95% CI: [24.0, 32.1]) than females (20.5%, 95% CI: [16.9, 24.6]), showing statistical significance (*p* = 0.036). The belief that AI will improve healthcare was shared by 72% (95% CI: [68.9, 74.8]) overall, though significantly more males (27.9%, 95% CI: [24.0, 32.1]) than females (15.4%, 95% CI: [12.3, 19.1]) thought AI would have no impact (*p* < 0.001). Most participants (83.8%, 95% CI: [81.2, 86.0]) reported prior AI use in practice, with significantly more males (88.1%, 95% CI: [84.8, 90.8]) than females (78.9%, 95% CI: [74.8, 82.6]), yielding a *p* value of < 0.001. Additionally, attitudes toward AI use in decision‐making were significantly different, with 17.9% (95% CI: [14.6, 21.9]) of females opposing its use entirely compared to only 7% (95% CI: [5.0, 9.7]) of males (*p* < 0.001).

**Table 4:** Gender differences in computer knowledge, AI experience, and perceptions of AI in healthcare.

	Total		Female		Male		*p* value
	col %	95% CI	col %	95% CI	col %	95% CI	
Rate your level of computer knowledge and expertise							
Not so familiar (*n* = 33)	3.7	[2.6, 5.1]	5.1	[3.4, 7.7]	2.3	[1.3, 4.2]	**0.022** *
Somewhat familiar (*n* = 374)	41.5	[38.3, 44.7]	43.6	[39.0, 48.3]	39.5	[35.2, 44.0]	
Very familiar (*n* = 495)	54.8	[51.6, 58.1]	51.3	[46.5, 56.0]	58.1	[53.6, 62.5]	
Any experience with AI							
No (*n* = 539)	59.8	[56.5, 62.9]	71.8	[67.3, 75.9]	48.8	[44.3, 53.3]	**<** **0.0001** *
Yes (*n* = 363)	40.2	[37.1, 43.5]	28.2	[24.1, 32.7]	51.2	[46.7, 55.7]	
Would you feel at ease using AI in your medical practice							
Not comfortable at all (*n* = 176)	19.5	[17.1, 22.2]	20.5	[16.9, 24.6]	18.6	[15.3, 22.4]	**0.036** *
Somewhat comfortable (*n* = 506)	56.1	[52.8, 59.3]	59	[54.2, 63.5]	53.5	[49.0, 58.0]	
Very comfortable (*n* = 220)	24.4	[21.7, 27.3]	20.5	[16.9, 24.6]	27.9	[24.0, 32.1]	
Believe AI can lead to better patient outcomes							
Agree (*n* = 341)	38.8	[35.6, 42.0]	36.8	[32.3, 41.6]	40.5	[36.1, 45.0]	0.532
Disagree (*n* = 88)	10	[8.2,12.2]	10.5	[7.9, 13.9]	9.5	[7.2, 12.6]	
Neither agree nor disagree (*n* = 451)	51.2	[47.9, 54.5]	52.6	[47.8, 57.4]	50	[45.4, 54.6]	
Do you see AI as having potential effects on the future of healthcare							
Have no impact (*n* = 198)	22	[19.4, 24.8]	15.4	[12.3, 19.1]	27.9	[24.0, 32.1]	**<** **0.001** *
Improve the future of healthcare (*n* = 649)	72	[68.9, 74.8]	71.8	[67.3, 75.9]	72.1	[67.9, 76.0]	
Worsen the future of healthcare (*n* = 55)	6.1	[4.7, 7.9]	12.8	[10.0, 16.3]	0		
You have not yet used AI in your healthcare practice							
No (*n* = 143)	16.3	[14.0, 18.8]	21.1	[17.4, 25.2]	11.9	[9.2, 15.2]	**<** **0.001** *
Yes (*n* = 737)	83.8	[81.2, 86.0]	78.9	[74.8, 82.6]	88.1	[84.8, 90.8]	
To what extent do you believe artificial intelligence (AI) should be used in making medical decisions for healthcare professionals?							
A little (*n* = 242)	26.8	[24.0, 29.8]	28.2	[24.1, 32.7]	25.6	[21.8, 29.7]	**<** **0.001** *
A lot (*n* = 154)	17.1	[14.8, 19.7]	12.8	[10.0, 16.3]	20.9	[17.5, 24.8]	
A moderate amount (*n* = 396)	43.9	[40.7, 47.2]	41	[36.5, 45.8]	46.5	[42.0, 51.0]	
None at all (*n* = 110)	12.2	[10.2, 14.5]	17.9	[14.6, 21.9]	7	[5.0, 9.7]	
This survey increased your desire to learn more about AI models							
No (*n* = 132)	14.6	[12.5, 17.1]	15.4	[12.3, 19.1]	14	[11.1, 17.4]	0.544
Yes (*n* = 770)	85.4	[82.9, 87.5]	84.6	[80.9, 87.7]	86	[82.6, 88.9]	

*Note:* Test statistics: *χ* ^2^ test.

* A *p* value less than 0.05 is considered statistically significant.

In Table 5, respondents with less than 5 years of experience reported the highest proportion of being “Not so familiar” with computers (4.3%, 95% CI: [2.4, 7.7]), while those with more than 20 years reported none (*p* = 0.001). AI experience also varied significantly, with the highest percentage of AI experience among those with 10–20 years (47.5%, 95% CI: [42.9, 52.2]) and the lowest among those with 5–10 years (33.3%, 95% CI: [26.6, 40.9]) (*p* = 0.001). Comfort in using AI in medical practice showed a strong association with experience (*p* < 0.0001), with 80% (95% CI: [73.2, 85.4]) of those with 5–10 years of experience being “Somewhat comfortable,” whereas 50% (95% CI: [30.2, 69.8]) of those with more than 20 years reported being “Not comfortable at all.” Belief in AI improving patient outcomes also showed significant differences (*p* < 0.0001), with only 26.7% (95% CI: [20.5, 33.9]) of those with 5–10 years agreeing, compared to 46.2% (95% CI: [41.5, 50.9]) of those with 10–20 years. Perceptions of AI's future impact on healthcare also differed (*p* < 0.0001); while 87% (95% CI: [82.2, 90.6]) of those with less than 5 years of experience believed AI would improve healthcare, only 60% (95% CI: [52.3, 67.2]) of those with 5–10 years shared this belief. Notably, AI utilization in practice did not significantly vary (*p* = 0.177). The extent to which AI should be used in medical decision‐making was significantly different (*p* < 0.0001), with 50% (95% CI: [30.2, 69.8]) of those with more than 20 years believing AI should be used “A lot,” compared to only 12.5% (95% CI: [9.7, 15.9]) of those with 10–20 years. Finally, 87.5% (95% CI: [85.1, 89.5]) of respondents overall expressed an increased desire to learn about AI, though this varied significantly by experience level (*p* < 0.0001), with 100% of those with 5–10 years showing interest, while only 50% (95% CI: [30.2, 69.8]) of those with more than 20 years expressed the same.

**Table 5:** Influence of years of medical experience on computer knowledge, AI experience, and perceptions of AI in healthcare.

	Total		10–20 years		5–10 years		Less than 5 years		More than 20 years		*p* value
	col %	95% CI	col %	95% CI	col %	95% CI	col %	95% CI	col %	95% CI	
Rate your level of computer knowledge and expertise											
Not so familiar (*n* = 33)	3.8	[2.7, 5.2]	2.5	[1.4, 4.5]	6.7	[3.7, 11.6]	4.3	[2.4, 7.7]	0		**0.001** *
Somewhat familiar (*n* = 352)	40	[36.8, 43.3]	42.5	[38.0, 47.2]	40	[32.8, 47.7]	39	[33.3, 45.3]	0		
Very familiar (*n* = 495)	56.3	[52.9, 59.5]	55	[50.3, 59.6]	53.3	[45.7, 60.8]	57	[50.3, 62.5]	100		
Any experience with AI											
No (*n* = 517)	58.8	[55.5, 62.0]	52.5	[47.8, 57.1]	66.7	[59.1, 73.4]	65	[59.1, 70.8]	50	[30.2, 69.8]	**0.001** *
Yes (*n* = 363)	41.3	[38.0, 44.5]	47.5	[42.9, 52.2]	33.3	[26.6, 40.9]	35	[29.2, 40.9]	50	[30.2, 69.8]	
Would you feel at ease using AI in your medical practice											
Not comfortable at all (*n* = 154)	17.5	[15.1, 20.2]	20	[16.5, 24.0]	6.7	[3.7, 11.6]	17	[13.2, 22.6]	50	[30.2, 69.8]	**<** **0.0001** *
Somewhat comfortable (*n* = 506)	57.5	[54.2, 60.7]	47.5	[42.9, 52.2]	80	[73.2, 85.4]	61	[54.7, 66.7]	50	[30.2, 69.8]	
Very comfortable (*n* = 220)	25	[22.2, 28.0]	32.5	[28.3, 37.0]	13.3	[8.9, 19.4]	22	[17.1, 27.3]	0		
Believe AI can lead to better patient outcomes											
Agree (*n* = 341)	39.7	[36.5, 43.1]	46.2	[41.5, 50.9]	26.7	[20.5, 33.9]	41	[34.9, 47.2]	0		**<** **0.0001** *
Disagree (*n* = 88)	10.3	[8.4, 12.5]	10.3	[7.7, 13.5]	20	[14.6, 26.8]	4.5	[2.5, 8.0]	0		
Neither agree nor disagree (*n* = 429)	50	[46.7, 53.3]	43.6	[39.0, 48.3]	53.3	[45.7, 60.8]	55	[48.2, 60.7]	100		
Do you see AI as having potential effects on the future of healthcare											
Have no impact (*n* = 187)	21.3	[18.7, 24.1]	22.5	[18.8, 26.6]	40	[32.8, 47.7]	4.3	[2.4, 7.7]	50	[30.2, 69.8]	**<** **0.0001** *
Improve the future of healthcare (*n* = 649)	73.8	[70.7, 76.6]	72.5	[68.1, 76.5]	60	[52.3, 67.2]	87	[82.2, 90.6]	50	[30.2, 69.8]	
Worsen the future of healthcare (*n* = 44)	5	[3.7, 6.7]	5	[3.3, 7.5]	0		8.7	[5.8, 12.9]	0		
You have not yet used AI in your healthcare practice											
No (*n* = 121)	14.1	[11.9, 16.6]	15.8	[12.6, 19.6]	13.3	[8.9, 19.4]	13	[9.4, 17.8]	0		0.177
Yes (*n* = 737)	85.9	[83.4, 88.1]	84.2	[80.4, 87.4]	86.7	[80.6, 91.1]	87	[82.2, 90.6]	100		
To what extent do you believe artificial intelligence (AI) should be used to make											
A little (*n* = 242)	27.5	[24.6, 30.6]	37.5	[33.1, 42.1]	20	[14.6, 26.8]	17	[13.2, 22.6]	0		**<** **0.0001** *
A lot (*n* = 154)	17.5	[15.1, 20.2]	12.5	[9.7, 15.9]	26.7	[20.5, 33.9]	17	[13.2, 22.6]	50	[30.2, 69.8]	
A moderate amount (*n* = 396)	45	[41.7, 48.3]	45	[40.4, 49.7]	40	[32.8, 47.7]	48	[41.7, 54.0]	50	[30.2, 69.8]	
None at all (*n* = 88)	10	[8.2, 12.2]	5	[3.3, 7.5]	13.3	[8.9, 19.4]	17	[13.2, 22.6]	0		
This survey increased your desire to learn more about AI models											
No (*n* = 110)	12.5	[10.5, 14.9]	17.5	[14.2, 21.3]	0		8.7	[5.8, 12.9]	50	[30.2, 69.8]	**<** **0.0001** *
Yes (*n* = 770)	87.5	[85.1, 89.5]	82.5	[78.7, 85.8]	100		91	[87.1, 94.2]	50	[30.2, 69.8]	

*Note:* Test statistics: *χ* ^2^ test

* A *p* value less than 0.05 is considered statistically significant.

## 4 Discussion

The findings of this study provide initial insights into HCWs' perceptions and use of AI in healthcare in Qatar. With a diverse sample of 902 participants, predominantly healthcare professionals, the study underscores the varied adoption and attitudes toward AI across different roles within healthcare settings. Additionally, the responses of HCWs indicate their readiness for AI implementation in healthcare and their belief in its potential to impact healthcare outcomes positively. These findings align with previous research that highlights HCWs' interest in utilizing AI to enhance patient outcomes [20, 21]. For example, Topol (2019) and Jiang et al. (2017) have highlighted AI's revolutionary potential in healthcare, notably in diagnostics and personalized medicine, which is consistent with our findings on HCWs' excitement about AI's role in enhancing clinical workflows [22, 23]. The results of this study not only contribute to understanding the current state of AI integration in healthcare but also provide a foundation for future research and development in this rapidly evolving field.

Many participants in this study reported using AI in their work. However, the relatively low usage of AI for medical decision‐making and supporting families and patients indicates significant room for improvement in these areas. This finding is consistent with previous literature, such as the work of Reddy et al. (2021), who found challenges to AI adoption in clinical decision‐making, including a lack of trust in AI outputs, insufficient training, and concerns about liability [24]. AI's potential to augment decision‐making and offer personalized support could be transformative, particularly in complex clinical scenarios and patient interactions [25]. To fill the gap, specialized initiatives like focused training programs, pilot projects demonstrating AI's efficacy in real‐world contexts, and legal frameworks addressing liability and ethical concerns could be deployed. For example, healthcare institutions may work with AI developers to provide user‐friendly interfaces and hands‐on training for HCWs, boosting trust in AI‐assisted decision‐making. For research purposes, the primary application of AI identified by respondents in this study was drafting manuscripts and assisting with scientific writing. Previous research has shown that AI can be an effective tool for improving the clarity, style, and coherence of scientific writing [17, 26]. This functionality highlights AI's potential to streamline administrative and documentation tasks, enabling healthcare professionals to allocate more time to direct patient care. Furthermore, AI's role in appraising medical literature and aiding in medical decision‐making underscores its capacity to enhance clinical efficiency and provide robust decision support. Accordingly, we can argue that AI's integration into clinical and research domains can significantly benefit healthcare outcomes by optimizing workflow and improving the quality of medical documentation and decision‐making processes.

However, many challenges of AI use should be highlighted, including privacy and data security [16, 27]. These concerns are reflected in the research, with Price and Cohen (2019) underlining the importance of strong data governance frameworks in protecting patient information in AI‐driven healthcare systems [28]. The perception of AI's capabilities remains moderate, with many participants expressing cautious optimism about its potential. This ambivalence may stem from factors such as the early stage of AI technology, concerns about reliability and accuracy, and a lack of comprehensive understanding of AI's capabilities among HCWs [29, 30, 31]. For example, Pumplun et al. (2021) discovered that HCWs' trust in AI is highly related to transparency in how AI algorithms operate and the capacity to check their results [32]. Moreover, there are potential risks associated with AI‐assisted scientific writing, including the possibility of errors or inconsistencies in research papers generated by AI systems and the risk of bias, given that AI models are trained on datasets that may contain biased or incomplete information [33]. Therefore, HCWs and researchers must be adequately trained in the use of AI to mitigate these risks and ensure the accuracy and integrity of AI‐assisted outputs. In line with Sommer et al.'s (2024) recommendations, we believe that educational campaigns and nurse involvement in AI development are critical steps toward increasing AI literacy and facilitating the successful integration of AI into nursing practice. These strategies can assist in overcoming the concerns and barriers raised by nurses, resulting in more effective AI applications in healthcare [10]. By enhancing education and awareness, the healthcare community can better harness AI's benefits while minimizing its potential drawbacks [31, 33].

The study also revealed a gender‐balanced sample, with nearly half of the participants being female. This gender distribution is crucial for understanding potential differences in perceptions and acceptance of AI technologies across different demographics. Recent research, such as that conducted by Leslie et al. (2021), has underlined the necessity of gender diversity in AI development and deployment to ensure equal outcomes and prevent reinforcing existing prejudices [34]. Additionally, the varied levels of comfort and confidence in using AI among healthcare professionals—such as nurses, physicians, pharmacists, and allied health professionals—highlight the need for tailored educational strategies. Future research could explore these profession‐specific attitudes toward AI to develop targeted strategies that address unique concerns and enhance overall acceptance.

### 4.1 Clinical Implication

The study on the perception and knowledge of hospital workers toward using AI in Qatar reveals critical insights with significant clinical implications. By identifying gaps in understanding and acceptance of AI, healthcare institutions can develop targeted training programs to enhance the diagnostic accuracy and efficiency of clinical workflows. For example, introducing AI literacy modules into continuing medical education (CME) programs could assist in closing the knowledge gap and increase acceptance of AI solutions. Educated and confident staff are more likely to utilize AI tools effectively, reducing human errors and improving patient safety. Moreover, the study highlights the importance of interdisciplinary collaboration and ethical considerations in AI implementation, ensuring that technological advancements complement rather than compromise the patient–doctor relationship. Policies that promote transparency in AI algorithms and establish clear criteria for AI use in clinical contexts may further boost confidence and adoption. Ultimately, these insights can guide the integration of AI in healthcare, fostering improved clinical outcomes and maintaining high standards of patient care.

### 4.2 Limitation

This study has limitations as a cross‐sectional study using a self‐reported questionnaire. Self‐reported data can be subject to biases, such as social desirability bias and recall bias, which may affect the accuracy of the responses. Podsakoff et al. (2003) found that participants may overreport their usage of AI due to perceived expectations or underreport concerns due to fear of criticism [35]. Additionally, the cross‐sectional design captures perceptions at a single point in time, limiting the ability to infer attitude and perception changes over time. To mitigate these limitations, pilot testing was carried out to modify the questionnaire and ensure clarity in the questions, thus lowering the risk of response bias. Future studies could benefit from longitudinal designs and objective measures of AI usage to provide a more comprehensive understanding of the integration of AI in healthcare. Furthermore, data triangulation may improve the validity of the findings by merging self‐reported data with observational or administrative data. A mixed methods approach, such as interviews or observational research, may also provide more detailed insights into the hurdles and facilitators of AI adoption in healthcare settings.

## 5 Future Research Directions

Future studies should concentrate on a few critical topics. Longitudinal studies are required to assess changes in HCWs' attitudes and uptake of AI over time as technology advances. Experimental approaches, such as randomized controlled trials, could be used to assess the efficacy of AI training interventions as well as their effects on HCWs' confidence and competence with AI tools. Research should also look into the impact of interdisciplinary collaboration in AI deployment, specifically how cooperation among healthcare practitioners, AI developers, and politicians affects AI acceptance. Furthermore, qualitative research, such as interviews or focus groups, may provide more in‐depth insights into the hurdles and facilitators of AI adoption from the perspective of diverse stakeholders. Addressing these issues would help to improve AI adoption in healthcare.

## 6 Conclusion

While AI usage among HCWs is evident and growing, there is a clear need for further education and confidence‐building measures. Addressing the moderate belief in AI's capabilities and expanding its applications beyond administrative tasks to more clinical roles will be crucial for maximizing the benefits of AI in healthcare. Healthcare systems can leverage these technologies to improve efficiency, accuracy, and patient outcomes by fostering a deeper understanding and trust in AI. However, it is vital to highlight that, due to the study's cross‐sectional methodology, causal links cannot be established. The findings show correlations rather than causality, and further research is needed to determine the long‐term effects of AI integration in healthcare.

## Author Contributions

**Ahmad R. Al‐Qudimat:** conceptualization, investigation, methodology, writing – review and editing, writing – original draft. **Mohammad R. Alqudimat:** methodology, writing – review and editing, writing – original draft. **Kalpana Singh:** writing – original draft, formal analysis. **Zainab E. Fares:** investigation, writing – original draft, data curation. **Mohammed Ismail:** investigation, data curation. **Hesham E. Yasin:** investigation, methodology, data curation. **Raed M. Al‐Zoubi:** writing – original draft, investigation. **Omar M. Aboumarzouk:** writing – original draft, supervision, writing – review and editing.

## Ethics Statement

The studies involving humans were approved by the Surgical Research Section, Hamad Medical Corporation, Qatar.

## Consent

Consent was obtained from all participants, who were informed about the study's objectives and assured that all shared information would remain confidential.

## Conflicts of Interest

The authors declare no conflicts of interest.

### 1 Transparency Statement

The lead author Ahmad R. Al‐Qudimat affirms that this manuscript is an honest, accurate, and transparent account of the study being reported; that no important aspects of the study have been omitted; and that any discrepancies from the study as planned (and, if relevant, registered) have been explained.

## Data Availability

The raw data supporting the conclusions of this article will be made available by the corresponding author upon request.
